# Immunohistological Expression of SOX-10 in Triple-Negative Breast Cancer: A Descriptive Analysis of 113 Samples

**DOI:** 10.3390/ijms21176407

**Published:** 2020-09-03

**Authors:** Katharina Kriegsmann, Christa Flechtenmacher, Jörg Heil, Jörg Kriegsmann, Gunhild Mechtersheimer, Sebastian Aulmann, Wilko Weichert, Hans-Peter Sinn, Mark Kriegsmann

**Affiliations:** 1 Department of Hematology, Oncology and Rheumatology, University Hospital Heidelberg, 69120 Heidelberg, Germany; Katharina.kriegsmann@med.uni-heidelberg.de; 2 Institute of Pathology, University Hospital Heidelberg, 69120 Heidelberg, Germany; christa.flechtenmacher@med.uni-heidelberg.de (C.F.); gunhild.mechtersheimer@med.uni-heidelberg.de (G.M.); peter.sinn@med.uni-heidelberg.de (H.-P.S.); 3 Breast Unit, Women’s Hospital, University of Heidelberg, 69120 Heidelberg, Germany; Joerg.heil@med.uni-heidelberg.de; 4 Institute of Pathology, Cytology and Molecular Pathology, 54296 Trier, Germany; kriegsmann@patho-trier.de; 5 Danube Private University Krems, 3500 Krems, Austria; 6 Optipath Institute of Pathology, 60311 Frankfurt, Germany; au@opti-path.de; 7 Institute of Pathology, TU Munich, 81675 Munich, Germany; wilko.weichert@tum.de

**Keywords:** SOX10, immunohistochemistry, triple-negative breast cancer

## Abstract

Background: SRY-related HMG-box 10 (SOX-10) is commonly expressed in triple negative breast cancer (TNBC). However, data on the biological significance of SOX-10 expression is limited. Therefore, we investigated immunhistological SOX-10 expression in TNBC and correlated the results with genetic alterations and clinical data. Methods: A tissue microarray including 113 TNBC cases was stained by SOX-10. Immunohistological data of AR, BCL2, CD117, p53 and Vimentin was available from a previous study. Semiconductor-based panel sequencing data including commonly altered breast cancer genes was also available from a previous investigation. SOX-10 expression was correlated with clinicopathological, immunohistochemical and genetic data. Results: SOX-10 was significantly associated with CD117 and Vimentin, but not with AR expression. An association of SOX-10 with BCL2, EGFR or p53 staining was not observed. SOX-10-positive tumors harbored more often TP53 mutations but less frequent mutations of PIK3CA or alterations of the PIK3K pathway. SOX-10 expression had no prognostic impact either on disease-free, distant disease-free, or overall survival. Conclusions: While there might be a value of SOX-10 as a differential diagnostic marker to identify metastases of TNBC, its biological role remains to be investigated.

## 1. Introduction

SRY-related HMG-box 10 (SOX-10) is a recently described molecule that regulates Wnt/β-catenin signaling, contributes to stem/progenitor activity and induces a mesenchymal transition expression [1,2]. SOX-10 immunohistochemistry (IHC) is commonly positive in melanoma [3,4], but has also been described in benign adnexal skin tumors such as cylindroma and spiradenoma (uniformly positive) [5]; in schwannoma [4]; in tumors of myoepithelial origin [4]; and in a subset of carcinomas such as bladder [6], breast [7], gastric [8], hepatocellular [9], nasopharyngeal [10], ovarian [11], prostate [12], as well as salivary gland tumors [13,14], and squamous cell carcinoma of head and neck [4]. Positivity is usually restricted to a small subset of carcinomas (<10%), except for triple-negative breast cancer (TNBC), which may more frequently express SOX-10 [4]. However, little is known of the biology of SOX-10-positive or -negative TNBC, and studies investigating SOX-10 expression are limited by a relatively low number of cases, lack of genetic data or lack of survival data. Therefore, we investigated immunohistological SOX-10 expression in a large cohort including 113 TNBC previously characterized by clinical and molecular data.

## 2. Results

### 2.1. SOX-10 Positivity Is Associated with Low Tumor Size at First Diagnosis

Overall, 113 TNBC cases were analyzed. Of these, 109 (96%) were classified as no special type and four (4%) as invasive lobular carcinoma according to the 4th Edition of the World Health Classification of Tumors of the Breast [15]. The median age at first diagnosis was 53 (28–90) years. Thirty six (32%), 53 (47%), 10 (9%) and 7 (6%) of patients were diagnosed with clinical stage I, II, III and IV respectively. The histopathological evaluation revealed high grading (grade 3) in most patients (*n* = 96, 95%). Neoadjuvant chemotherapy was administered to 20 (18%) patients.

**Figure 1:**
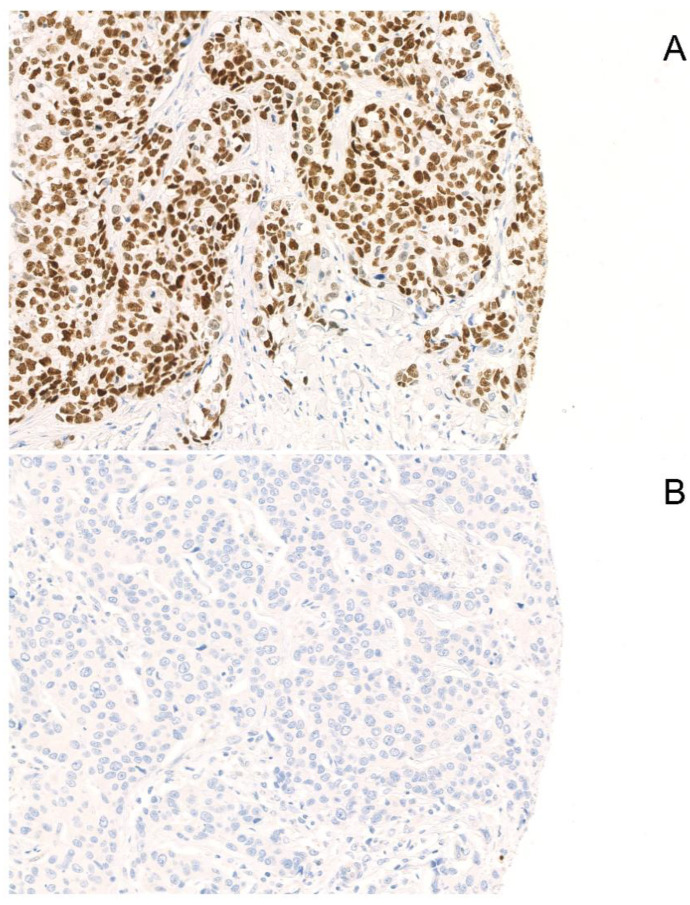
Examples of SOX-10-positive and -negative staining. A typical example of positive (**A**) and negative (**B**) nuclear staining of SOX-10 is displayed (Magnification: 200×).

SOX-10 positivity was found in 46 (41%) patients, while 67 (59%) patients were negative. Typical examples of positive and negative staining are displayed in Figure 1.

Comparing patients’ clinical characteristics at first diagnosis, statistically significant difference was identified regarding tumor size. In particular, SOX-10 positivity was associated with a higher proportion of pT1 tumors (*n* = 24, 52%), compared to SOX-10-negative cases (*n* = 22, 33%, *p* = 0.039). Patients’ characteristics for the overall cohort and regarding SOX-10 expression are shown in Table 1.

### 2.2. SOX-10-Positive Cases Show a Higher Proportion of CD117 and Vimentin Positivity

IHC evaluation of other antigens (androgen receptor (AR), B-cell lymphoma 2 (BCL2), cluster of differentiation (CD)117, epidermal growth factor receptor (EGFR), p53 and Vimentin) was performed in order to identify antigen patterns associated with SOX-10 positivity or negativity.

AR positivity was rare, and was found in 8 (7%) patients only. BCL2 and CD117 were identified in 16 (14%) and 18 (16%) cases, respectively. In the whole cohort, 40 (35%) patients were positive for EGFR. p53 overexpression (or absence) was identified in 56 (50%) patients. Vimentin positivity was found in 44 (39%) patients.

Interestingly, differences in CD117 and vimentin expression were found regarding SOX-10 expression. The proportion of CD117 positive cases was significantly higher in SOX-10-positive cases (*n* = 12, 26%), compared to SOX-10-negative cases (*n* = 6, 9%, *p* = 0.012). Similarly, the proportion of vimentin positive cases was significantly higher in SOX-10-positive cases (*n* = 31, 67%), compared to SOX-10-negative cases (*n* = 13, 19%, *p* < 0.001). IHC characteristics with regard to SOX-10 expression are displayed in Table 2.

### 2.3. Higher Proportion of TP53 Mutations and Lower Proportion of PIK3K Pathway Alterations in SOX-10-Positive Patients

Data from sequencing analysis with regard to mutations/alterations in TP53; PIK3CA; as well as PIK3K-, cell cycle- and MAPK-pathways was available for 73 patients. Overall, TP53 and PIK3CA mutations were found in 61 (84%) and 17 (23%) cases, respectively. Alterations in PIK3K-, cell cycle- and MAPK-pathways were evident in 20 (27%), 13 (18%) and 6 (8%) patients, respectively. Interestingly, the proportion of TP53 mutations was higher in SOX-10-positive cases (*n* = 32, 97%), compared to SOX-10-negative cases (*n* = 29, 73%, *p* = 0.005). On the contrary, PIK3K pathway alterations, including PIK3CA mutations, were less frequent in SOX-10-positive (*n* = 3, 9%) than -negative cases (*n* = 17, 43%, *p* = 0.001). An overview on molecular genetic characteristics is given in Table 3.

### 2.4. Survival Analysis

**Figure 2:**
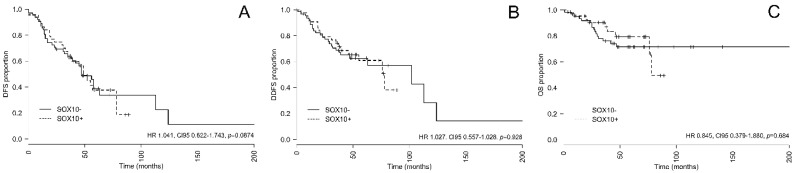
Survival analysis with regard to SOX-10 status. The figure shows Kaplan–Meier curves for (**A**) disease-free survival (DFS), (**B**) distant disease-free survival (DDFS), and (**C**) overall survival (OS) for SOX-10-positive and -negative patients. HR, Hazard ratio; CI, confidence interval; SOX-10, SRY-related HMG-box 10.

To evaluate the prognostic significance of SOX-10, survival analyses were performed. Sixty (53%) of 113 analyzed patients experienced a relapse. Twenty five (42%) of these occurred in SOX-10-positive and 35 (58%) in SOX-10-negative patients, respectively. The median disease-free survival (DFS) was 49 and 47 months in SOX-10-positive and -negative patients. Neither in univariate (Hazard ratio (HR) 1.041, CI_95_ 0.622–1.743, *p* = 0.874, Figure 2A) nor in multivariate (HR 1.168, CI_95_ 0.692–1.970, *p* = 0.562, Table 4) analysis was a statistically significant difference with regard to DFS was observed between SOX-10-positive and -negative patients.

Distant recurrence of disease was observed in 43 (38%) of patients. Seventeen (37%) distant relapses occurred in SOX-10-positive and 26 (39%) in SOX-10-negative patients, respectively. The median distant disease-free survival (DDFS) was 78 and 102 months in SOX-10-positive and -negative patients, respectively. Neither on univariate (HR 1.027, CI_95_ 0.557–1.028, *p* = 0.928, Figure 2B) nor on multivariate (HR 1.078, CI_95_ 0.574–2.023, *p* = 0.815, Table 4) analysis a statistically significant difference with regard to DDFS was observed between SOX-10-positive and -negative patients.

Overall, 25 (22%) deaths occurred. Nine (20%) patients died in the SOX-10-positive and 16 (24%) in SOX-10-negative subgroup, respectively. The median overall survival (OS) was 78 in SOX-10-positive and not reached in SOX-10-negative patients. Neither in univariate (HR 0.845, CI_95_ 0.379–1.880, *p* = 0.684, Figure 2C) nor multivariate (HR 0.865, CI_95_ 0.380–1.969, *p* = 0.730, Table 4) analysis was a statistically significant difference regarding OS observed between SOX-10-positive and -negative patients.

The only variable with prognostic relevance on multivariate analysis was clinical stage at first diagnosis. Higher stage was a parameter for poor prognosis regarding DFS, DDFS and OS. Neither age nor grading was statistically relevant for outcome in the investigated cohort (Table 4). Including neoadjuvant chemotherapy as an additional variable into the multivariate survival analysis did not significantly change the results and was not a statistically significant predictor for DFS, DDFS or OS.

## 3. Discussion

In the present study, we evaluated 113 TNBC cases, which was among the largest TNBC cohort analyzing SOX-10 immunohistological expression to date. Overall, we found SOX-10 positivity in 41% of TNBC, which was within the reported range of 38–67% [16,17,18].

Regarding clinical variables SOX-10 showed a tendency towards smaller tumors in the current analysis. Although more SOX-10-positive pT1 tumors were observed than pT2 tumors, in prior studies, this difference was not significant, which may have been due to the limited sample size in other investigations [16]. As in other studies, no association was identified regarding age, nodal involvement or other clinicopathological variables [16].

We correlated SOX-10 expression with immunohistochemical staining results previously acquired and observed that SOX-10-positive tumors do not label with AR, which has also been observed by other investigators [16]. SOX-10 was associated with Vimentin and CD117 expression, both markers that have been linked to worse survival in TNBC [19,20]. The co-expression of SOX-10 and Vimentin support the idea that SOX-10-positive tumors have a mesenchymal phenotype [21]. No association between SOX-10 and BCL2, EGFR and p53 was observed in our study. Lack of an association between SOX-10 and EGFR is in line with the literature, while BCL2 and p53 were not studied in this context [16].

Immunohistochemical SOX-10 expression has not been linked to genetic alterations in TNBC so far. In this context, it is important to note that TP53 mutations are more common in TNBC than luminal or HER2 phenotypes supporting the role of TP53 mutations as the key genetic event in this population [22]. Moreover, alterations of the PIK3 pathway are common findings, with frequencies reported in TNBC of up to 30%, composed mainly of PIK3CA mutations (approximately 22%) [23,24]. BRCA gene mutations, which are also frequently observed in TNBC, were not included in our panel [25]. Therefore, we could not correlate SOX-10 expression with the BRCA mutational status. We observed a higher proportion of *TP53* mutations and a lower proportion of PIK3K pathway alterations in SOX-10-positive tumors. However, the significance of this finding remains to be investigated. When interpreting the results of immunohistochemical SOX-10 expression, it is important to note that point mutations and copy number aberrations of the SOX-10 gene in form of amplifications and homozygous deletions have been reported in breast cancer and may lead to overexpression and lack of expression of SOX-10 [26]. Thus, a negative staining result may not necessarily indicate that alterations on the genetic level of SOX-10 are absent.

As in previous studies, an effect of SOX-10 expression on OS, DDFS or DFS could not be demonstrated [16,18,27].

Recent evidence suggests a potential role of SOX-10 in the differential diagnostic context to identify metastatic TNBC [28]. GATA binding protein 3 (GATA3) is a commonly used sensitive marker to identify mammary origin of metastatic TNBC. In this regard, SOX-10 has been reported to be commonly positive in GATA3-negative TNBC metastases [28]. The combination of SOX-10 and GATA3 yielded a sensitivity of about 60% for the identification of TNBC in a recent study investigating >1800 cases [29]. The combination of GATA3 and SOX-10 staining has been proven to be useful to identify mammary origin in several previous studies [30,31,32,33].

## 4. Materials and Methods

### 4.1. Patient Characteristics

Our tissue cohort consisted of 113 cases of TNBC samples. Diagnoses were made according to the World Health Organization classification of tumors of the breast [15]. Negative estrogen-, progesteron and human epidermal growth factor receptor 2 (HER2/(neu)) receptor status of the tumors was defined according to current guidelines recommendations from the American Society of Clinical Oncology/College of American Pathologists [34,35]. The samples were provided by the tissue bank of the National Center for Tumor Diseases (NCT, Heidelberg, Germany) in accordance with the local regulations and the approval of the ethics committee of the University of Heidelberg (#2463 and #S315-2020). All patients were diagnosed and treated between 2003 and 2006. Clinicopathological characteristics of the cohort are summarized in Table 1.

### 4.2. Immunohistochemistry

Immunohistochemical staining was performed as previously described [36]. In brief, slides were deparaffinized, pre-treated with an antigen retrieval buffer and stained using an automated device. All antibody stainings were carried out on a Ventana Benchmark Ultra (Roche, Rotkreuz, Switzerland). The antibody and staining characteristics are shown in Supplementary Table S1. Immunohistochemical stains other than SOX-10 were available from a previous study [37]. Among them are AR, BCL2, CD117 and the mesenchymal intermediary filament vimentin, which have all been reported in subgroups of TNBC [37].

### 4.3. Semiconductor-Based Panel Sequencing

Sequencing was performed as previously described using a custom-made breast cancer panel in combination with multiplex polymerase chain reaction-based Ion Torrent AmpliSeqTM technology (Thermo Fisher scientific, Waltham, MA, USA) [24]. Results from these analyses were previously published [24]. Genes included in the breast cancer panel are outlined in Supplementary Table S2. *TP53* and *PIK3CA* were analyzed as single genes. *PIK3CA* mutations, *PIK3CA* amplifications, *PTEN* mutations, *PIK3R1* mutations and *AKT1* mutations were combined as PIK3K-pathway alterations. *RB* mutations, *RB* deletions, *CDKN2A* mutations, *CDKN1B* mutations and *CDK4* mutations were combined as molecular alterations of the cell cycle pathway. Finally, *MAP2K4* amplifications, *HER2* mutations, *EGFR* mutations and *KRAS* mutations were combined as MAPK-pathway alterations. As no alterations in other genes were detected in our cohort, only the previously mentioned genes/subgroups were further analyzed.

### 4.4. Statistical Analysis

Statistical analysis was performed with R (v. 3.6.0, R Development Core Team, 2008). For descriptive statistics, data are presented as absolute numbers and percentages, and as the median and range. For comparison of categorical variables, Chi-square test in cases of 2 × 2 contingency tables or its extension in cases of 2x > 2 contingency tables were used. To identify differences among groups in cases of continuous variables, a two-sided independent t-test was performed. Survival was calculated and plotted using Kaplan–Meier survival analysis. To calculate the differences between the survival curves, a log-rank test was used. The Cox proportional hazard model and the Breslow method were applied for multivariate analysis. A *p* value < 0.05 was considered significant.

## 5. Conclusions

In summary, SOX-10 was significantly associated with CD117 and Vimentin, but not with AR expression. SOX-10-positive tumors harbored more often TP53 mutations but fewer frequent mutations of PIK3CA or alterations of the PIK3K pathway. SOX-10 expression had no prognostic impact either on DFS, DDFS or OS. Additional research is needed to understand the biological role of SOX-10 in TNBC.

## Figures and Tables

**Table 1:** Patients characteristics for the overall cohort and with regard to SOX-10 expression.

Variables	Overall Cohort	SOX-10		*p* Value
		Positive	Negative	
Overall cases, *n* (%)	113 (100)	46 (41)	67 (59)	/
Age at first diagnosis, median years (min–max)	53 (28–90)	53 (31–83)	58 (28–90)	0.221
Subtype, *n* (%)				n.a.
No special type (NST)	109 (96)	44 (96)	65 (97)	
Invasive lobular carcinoma (ILC)	4 (4)	2 (4)	2 (3)	
T stage, *n* (%)				**0.039 ^1^**
pT1	46 (41)	24 (52)	22 (33)	
pT2	54 (48)	17 (37)	37 (55)	
pT3	6 (5)	3 (7)	3 (4)	
pT4	7 (6)	2 (4)	5 (8)	
N stage, *n* (%)				0.407 ^2^
pN0	66 (58)	29 (63)	37 (55)	
pN1	22 (19)	9 (20)	13 (19)	
pN2	13 (12)	4 (9)	9 (13)	
pN3	10 (9)	3 (7)	7 (10)	
pNX	2 (2)	1 (2)	1 (1)	
Clinical stage, *n* (%)				0.385 ^3^
I	36 (32)	18 (39)	18 (27)	
II	53 (47)	19 (41)	34 (51)	
III	17 (15)	6 (13)	11 (16)	
IV	7 (6)	3 (7)	4 (6)	
Grading				0.622 ^4^
1	1 (1)	0 (0)	1 (1)	
2	16 (14)	6 (13)	10 (15)	
3	96 (95)	40 (87)	56 (84)	
Lymphangiosis, *n* (%)				0.093
yes	50 (44)	16 (35)	34 (51)	
no	63 (56)	30 (65)	33 (49)	
Lymphocytic stroma, *n* (%)				0.741 ^5^
absent	18 (16)	6 (13)	12 (18)	
mild	34 (30)	15 (33)	19 (28)	
prominent	57 (50)	24 (52)	33 (49)	
NA	4 (4)	1 (2)	3 (5)	

n.a., not appliacable; NA, not available; SOX-10, SOX-10, SRY-related HMG-box 10. ^1^ pT1 vs. ≥pT2; ^2^ pN0 vs. ≥pN1; ^3^ I vs. II vs. III/IV; ^4^ 1/2 vs. 3; ^5^ NA not included. Significant values are highlighted in bold.

**Table 2:** Immunohistochemical characteristics with regard to SOX-10 expression.

Variable	Overall Cohort	SOX-10		*p* Value *
		Positive	Negative	
Overall cases, *n* (%)	113 (100)	46 (41)	67 (59)	
AR, *n* (%)				n.a.
positive	8 (7)	0 (0)	8 (12)	
negative	105 (93)	46 (100)	59 (88)	
BCL2, *n* (%)				0.778
positive	16 (14)	6 (13)	10 (15)	
negative	97 (86)	40 (97)	57 (85)	
CD117, *n* (%)				**0.012**
positive	18 (16)	12 (26)	6 (9)	
negative	92 (81)	32 (70)	60 (90)	
NA	3 (3)	2 (4)	1 (1)	
EGFR, *n* (%)				0.101
positive	40 (35)	12 (26)	28 (42)	
negative	72 (64)	33 (72)	39 (58)	
NA	1 (1)	1 (2)	0 (0)	
p53, *n* (%)				0.219
positive	56 (50)	26 (57)	30 (45)	
negative	57 (50)	20 (43)	37 (55)	
Vimentin, *n* (%)				**<0.001**
positive	44 (39)	31 (67)	13 (19)	
negative	66 (58)	13 (28)	53 (79)	
NA	3 (3)	2 (4)	1 (1)	

AR, androgen receptor; BCL2, B-cell lymphoma 2; CD, cluster of differentiation; EGFR, epidermal growth factor receptor; n.a., not applicable; NA, not available, SOX-10, SRY-related HMG-box 10. * NA cases not included. Significant values are highlighted in bold.

**Table 3:** Molecular genetic characteristics with regard to SOX-10 expression.

Variable	Overall Cohort ^#^	SOX-10 ^#^		*p* Value *
		Positive	Negative	
Overall cases, *n* (%)	113 (100)	46 (41)	67 (59)	/
TP53, *n* (%)				**0.005**
mutated	61 (54/84)	32 (70/97)	29 (43/73)	
wild type	12 (11/16)	1 (2/3)	11 (16/28)	
NA	40 (35)	13 (28)	27 (40)	
PIK3CA, *n* (%)				**0.002**
mutated	17 (15/23)	2 (4/6)	15 (22/38)	
wild type	56 (50/77)	31 (67/94)	25 (37/63)	
NA	40 (35)	13 (28)	27 (40)	
PIK3K pathway, *n* (%)				**0.001**
altered	20 (18/27)	3 (7/9)	17 (25/43)	
not altered	53 (47/73)	30 (65/91)	23 (34/58)	
NA	40 (35)	13 (28)	27 (40)	
Cell cycle pathway, *n* (%)				0.249
altered	13 (12/18)	4 (9/12)	9 (13/23)	
not altered	60 (53/82)	29 (63/88)	31 (46/78)	
NA	40 (35)	13 (28)	27 (40)	
MAPK pathway, *n* (%)				0.805
altered	6 (5/8)	3 (7/9)	3 (4/8)	
not altered	67 (59/92)	30 (65/91)	37 (55/93)	
NA	40 (35)	13 (28)	27 (40)	

SOX-10, SRY-related HMG-box 10. ^#^ *n* absolute (% of all cases/% of available cases); * NA cases not included. Significant values are highlighted in bold.

**Table 4:** Multivariate survival analysis.

Variable	DFS		DDFS		OS	
	HR (CI_95_)	*p* Value	HR (CI_95_)	*p* Value	HR (CI_95_)	*p* Value
Age (<50 years vs. ≥50 years)	1.124 (0.653–0.1935)	0.673	1.075 (0.554–2.085)	0.830	0.729 (0.316–1.683)	0.459
Stage (I–IV)	2.428 (1.747–3.374)	**<0.001**	2.111 (1.479–3.013)	**<0.001**	3.172 (2.042–4.929)	**<0.001**
Grading (G1 */G2 vs. G3)	2.289 (0.693–7.556)	0.174	1.383 (0.402–4.760)	0.607	0.802 (0.173–3.730)	0.779
SOX-10 (negative vs. positive)	1.168 (0.692–1.970)	0.562	1.078 (0.574–2.023)	0.815	0.865 (0.380–1.969)	0.730

Ctx, chemotherapy; DFS, disease-free survival; DDFS, distant disease-free survival; OS, overall survival; SOX-10, SRY-related HMG-box 10. * G1 *n* = 1. Significant values are highlighted in bold.

## Supplementary Materials

The following are available online at https://www.mdpi.com/1422-0067/21/17/6407/s1.
