# Supplementary material for: Immunohistological Expression of SOX-10 in Triple-Negative Breast Cancer: A Descriptive Analysis of 113 Samples
Source: Int J Mol Sci. 2020 Sep 3;21(17):6407. doi: 10.3390/ijms21176407 (PMC7503807; doi:10.3390/ijms21176407)
Supplement: Supplementary file 1 [file ijms-21-06407-s001.zip › ijms-897535-supplementary.docx]

**Supplementary Table 1: Antibody characteristics and staining conditions.**

| **Antibody** | **Company** | **Clone** | **Pretreatment** | **Buffer incubation time**  **(min)** | **Antibody incubation time (min)** | **Dilution** |
| --- | --- | --- | --- | --- | --- | --- |
| AR | Dako | AR441 | Tris/Borat/EDTA, pH 8.4 | 40 | 24 | 1:25 |
| BCL2 | Dako | 124 | Citrate, pH 6.0 | 10 | 30 | 1:75 |
| CD117 | Dako | c-Kit | Citrate, pH 6.0 | 10 | 30 | 1:50 |
| EGFR | EmergoEurope | 31G7 | Protease I | 8 | 28 | 1:25 |
| p53 | Dako | DO-7 | EDTA, pH 9.0 | 10 | 30 | 1:100 |
| SOX-10 | Cell Marque | 383R-15 | Tris/Borat/EDTA, pH 8.4 | 48 | 24 | 1:100 |
| Vimentin | Dako | V9 | Citrate, pH 6.0 | 10 | 30 | 1:2000 |

AR, androgen receptor; BCL2, B-cell lymphoma 2; CD, cluster of differentiation; EDTA, Ethylenediaminetetraacetic acid, EGFR, epidermal growth factor receptor; SOX-10, SRY-related HMG-box 10

**Supplementary Table 2: Genes included in the sequencing panel.**

| **Genes in Breast Cancer Panel** | | | | |
| --- | --- | --- | --- | --- |
| AFF2  2 | CDKN1B  1 | KRAS  2,3 | PIK3R1  10,11,13 | TLR4  4 |
| AKT1  3 | CDKNA2  1,2 | MAP2K4  3,5,7-9 | PTEN  1,3,5-8 | TP53  4-10 |
| APC  16 | CEP164  13 | MAP3K1  4,9,10,13,14,17-20 | PTPRD  18,23,34 | USP36  17 |
| ARID1A  20 | CTCF  3,4 | MDM2  4,11 | RB1  2,3,6,13,16-18,20-23 | ZNF703  1 |
| BRAF  15 | EGFR  18-21 | MLL3  7,14,23,43 | RBMX  4 |  |
| CASP8  3,9 | ERBB2  19-21 | MYC  2,3 | RPS6KA1  11,14 |  |
| CBFB  3,4 | FGFR1  5,14 | NOTCH1  34 | RUNX1  5,7,8 |  |
| CCND1  1,3 | GATA3  5,6 | NR1H2  6 | SF3B1  14,15 |  |
| CDH1  2-7, 9-14, 16 | GIGYF2  29 | PAK1  2,13 | TBL1XR1  5 |  |
| CDK4  4,7 | HERC1  27 | PIK3CA  2,5,8,10,14,21 | TBX3  1,2 |  |

Exons included are given below each gene.
